# Fruit fly for anticancer drug discovery and repurposing

**DOI:** 10.1097/MS9.0000000000000222

**Published:** 2023-02-07

**Authors:** Firzan Nainu, Emil Salim, Muh. Fadhil As’ad, Deepak Chandran, Kuldeep Dhama, Ali A. Rabaan, Talha B. Emran

**Affiliations:** a Department of Pharmacy, Faculty of Pharmacy, Hasanuddin University, Tamalanrea, Makassar; b Department of Pharmacology, Faculty of Pharmacy, Universitas Sumatera Utara, Medan; c Graduate School of Pharmacy, Faculty of Pharmacy, Hasanuddin University, Tamalanrea, Makassar, Indonesia; d Department of Veterinary Sciences and Animal Husbandry, Amrita School of Agricultural Sciences, Amrita Vishwa Vidyapeetham University, Coimbatore, Tamil Nadu, India; e Division of Pathology, ICAR-Indian Veterinary Research Institute, Izatnagar, Bareilly, Uttar Pradesh, India; f Molecular Diagnostic Laboratory, Johns Hopkins Aramco Healthcare, Dhahran, Saudi Arabia; g College of Medicine, Alfaisal University, Riyadh, Saudi Arabia; h Department of Pharmacy, Faculty of Allied Health Sciences, Daffodil International University, Dhaka, Bangladesh; i Department of Pharmacy, BGC Trust University Bangladesh, Chittagong, Bangladesh

*Dear Editor*,

In 2020, around one of six deaths was attributed to cancer-related diseases, making cancer as one of the leading causes of mortality worldwide^1^. Cancer is a complex multifaceted disease, predominantly dictated by uncontrolled transformation of normal healthy cells into tumorous unwanted cells in a multistage process, called tumorigenesis. Considerable advances have been made to reveal predisposing factors and mechanisms of tumorigenesis, genetic alterations responsible in the emergence of malignant tumors, diagnostic features, and pharmacological options feasible in the treatment of cancer^2^. Nevertheless, despite such advances, classical problems remain the same: low rates of success in cancer drug discovery accompanied by inconvenient toxicological properties and high risk of cancer resistance against the currently available anticancer drugs^2^,^3^. Hence, alternative approaches are desperately required to achieve a better outcome in the clinical management of patients with cancer.

To address such issues, comprehensive screening of cancer drug candidates was introduced, aiming to find suitable candidates with effective dose to treat cancer but with less side effects^4^. However, in the in-vivo context, preclinical mammalian models are difficult to be used as a high-throughput screening platform due to economical and ethical issues^5^. To increase the chance to obtain prospective anticancer drug candidates with desired properties, the use of fruit fly *Drosophila melanogaster* as an in-vivo preclinical model has been introduced. With its high genetic similarity to humans, this insect is highly believed to provide a robust high-throughput in-vivo screening platform before thorough investigation using the mammalian model and eventually clinical testing in humans^6^.

Can we grow tumors in *Drosophila*? This question is simple but retains the most important aspect to determine the feasibility of *Drosophila* in cancer research. While some remains skeptical about this, evidence suggests that tumor cells can indeed grow in the fruit flies, inducible either by knocking down tumor suppressor genes or by manipulating pathways that are responsible for the induction of cancer^7^,^8^. Methods on how to perform such experiments can be found elsewhere and excellently compiled in the study by Gonzalez and colleagues^7^,^8^. Thus, we will not describe such topics exhaustively in this opinion piece. Based on the origin of tumorous cells, tumor cells studied in the fly models of cancer may arise from three different approaches: naturally grown tumor, genetic manipulation in the fly genes to induce cell transformation leading to tumor, and patient-derived tumor xenografts^7^. However, taking aside the differences of each approach, it is important to note that numerous signaling pathways responsible in the emergence of tumor cells in human, surprisingly, also play similar roles in the tumor development in *Drosophila* 9.

At present, the most common way to construct tumor-bearing flies is through overexpression of tumor-promoting genes or via suppression of tumor-inhibitory genes. This has been exemplified by several studies. In one study, the authors constructed tumor-bearing flies by promoting simultaneous inhibition of cell cycle checkpoints and apoptosis^10^, based on the protocol developed by other researchers^11^. Alternatively, tumor cells can be induced in flies by overexpressing oncogenic protein such as Ras (Ras^v12^)^12^ or by carrying out allograft method to implant tumorous larval tissue into the adult fly host^13^. With its simplicity and tractability for genetic manipulation^14^,^15^, *D. melanogaster* shall offer a rapid and robust yet economical approach to investigate the role of certain genetic manipulations in the tumor development as well as its pharmacological interventions. In addition, using *Drosophila* model, it was demonstrated that Myc-overexpressing cancer cells increased the growth of tumor by eliminating the surrounding healthy cells, a phenomenon called as cell competition, which has made outstanding contributions to cancer research by establishing fundamental knowledge on the mechanisms and genes involved in the growth control^16^. Certainly, this model has provided hints on the conserved mechanisms responsible in the cell transformation leading to the arise of tumor cells as well as antitumor innate immunity activated or repressed in the metazoan host during the tumor development (Table 1).

**Table 1:** Examples of anticancer drugs and prospective anticancer candidates tested using *Drosophila* model

Anticancer drugs/compounds	Target/pathways	Experimental systems	Lessons learned from *Drosophila* model	References
AUH-6-96	JAK-STAT pathway	*In vitro*, *Drosophila* cell culture Drug dose: 40 μmol/l	AUH-6-96 is a novel inhibitor of JAK/STAT signaling, which may have prospective pharmacotherapeutic role in the treatment of cancer in humans	17
Acivicin	CTP synthase	*In vivo*, larvae and adult *Drosophila* Drug dose: 10 mM	CTP synthase is an important target of acivicin-mediated inhibition in *Drosophila*	18
Afatinib, gefitinib, ibrutinib, bazedoxifene and afatinib	EGFR	*In vivo, Drosophila* larvae with ectopic expression of constitutively active EGFR (EGFR^CA^) in the airway system Drug doses: 50 µmol/l	Establishment of *Drosophila* lung cancer model by ectopic expression of constitutively active EGFR isoform (EGFR^CA^) in the airway system. Several drugs, in single or combination, were identified using this screening platform	19
Amsacrine	DNA-Topo II complex	*In vitro*, *Drosophila* cell culture Drug doses: 1, 2.5 mM and in combination with EMS	Homologous pairs can be negatively affected through the disrupted function of the *Drosophila* topoisomerase II (Top2) gene by RNAi and chemical inhibitors such as amsacrine	20,21
Artemisinin, curcumin	ROS	*In vivo, Drosophila* third instar larvae, l(2)gl-deficient line Drug dose: artemisinin (500 μM) and curcumin (100 μM)	Artemisinin and curcumin exhibited anticancer activity against brain tumors via the production of ROS	22
AY9944	Hh signaling	*In vivo, Drosophila* larvae (GAL4-UAS system) Drug doses: 3–30 μM	Insight on the mechanism of AY9944 and potential role of cholesterol transporters in the Hh signaling	23
BOT-4-one	JAK-STAT pathway	*In vitro*, *Drosophila* cell culture Drug dose: 30 μM	BOT-4-one is a novel inhibitor of JAK/STAT signaling, which may have prospective pharmacotherapeutic role in the treatment of cancer in humans	24
Bouvardin	Elongation step in protein synthesis	*In vivo, Drosophila* larvae Drug dose: 16 μM	Bouvardin was reported to inhibit the translation elongation as an enhancer of radiotherapeutic and chemotherapeutic agents	25
Cisplatin, cyclophosphamide	DNA alkylation and crosslinking	*In vivo, Drosophila* third instar larvae Drug doses: 50, 100, 200 μg/ml	Validation of neutral comet assay as an *in vivo* method to examine anticancer drug-genotoxicity in *Drosophila melanogaster*	26
Cobimetinib	dRaf	*In vivo, Drosophila* larvae (GAL4-UAS system) Drug dose: 200 μM	MEK1/2 inhibitors such as cobimetinib can be validated in an experimental setting using a *Drosophila* model	27
Cpd E, cyclopiazonic acid, DAPT, thapsigargin	Stem cell differentiation	*In vivo*, adult *Drosophila* Drug doses: cpd E (100 µM), cyclopiazonic acid (1 mM), DAPT (400 µM), and thapsigargin (100 µM)	Identification and validation of SERCA as a drug target for treating Notch1-related cancer. Inhibition of SERCA preferentially impairs maturation and activity of the mutant Notch1 receptor and induces G0/G1 arrest in NOTCH1 mutant cells	28
Crizotinib	hALK	*In vivo, Drosophila* larvae (GAL4-UAS system) Drug dose: 1 μM	Crizotinib is an anticancer drug with promising activity for hTPM4-hALK expressing cancers	29
Docetaxel, paclitaxel	Microtubule	*In vivo, Drosophila* larvae Drug doses: 0.0001–0.01 mM	Docetaxel and paclitaxel can be used in treatment of various types of cancer. While both drugs main cellular targets are alpha and beta tubulin dimers, paclitaxel was more nongenotoxic compared with docetaxel	30
Doxorubicin, idarubicin	DNA-Topo II complex	*In vivo, Drosophila* larvae Drug doses: 0.25, 0.5, 1, and 2.5 mM	The genotoxic effect of anthracycline compounds such as doxorubicin and idarubicin are due to their stabilizing effect on the Topo II-cleaved DNA complex, inducing high frequency of homologs recombination	31,32
F14512	DNA-Topo II complex	*In vitro*, *Drosophila* cell culture *In vivo, Drosophila* larvae Drug doses: 1, 5, 25, 50, and 100 μM	F14512 is a novel anticancer compound with antiproliferative activity in *Drosophila*. The anticancer effect of F14512 was suggested based on its ability to stabilize Topo II-cleaved DNA complex	33
Gefitinib and erlotinib	EGFR, ERK	*In vivo*, adult *Drosophila* (GAL4-UAS system) Drug doses: 25 and 200 ppm	Inhibitors of tyrosine kinase can act as new anticancer drugs	34
2HMC and 4-MET	DNA	*In vivo, Drosophila* third instar larvae Drug doses: 1, 5, 10, 50, 100, 200, and 400 μg/ml	2HMC and 4-MET are chalcones with prospective anticancer effect. These compounds were not toxic to the *Drosophila* model	35
Indomethacin	hAPC, Wnt/Wg signaling pathway	*In vivo*, adult *Drosophila* (GAL4-UAS system) Drug dose: 200 mg/ml in fly food	Indomethacin acts in the *Drosophila* Wnt/Wg pathway. Indomethacin specifically enhances the hAPC-induced phenotype	36
Imipramine	Fascin pathway	*In vitro*, *Drosophila* cell culture Drug dose: 10 μM	*Drosophila* may serve as a prospective model for drug repurposing approach, especially in the investigation of fascin pathway modulators. Fascin is one of important players in the tumor invasion and metastasis	37
Methotrexate, aminopterin	JAK-STAT pathway	*In vivo, Drosophila* larvae Drug dose: NA (article is a poster abstract)	Methotrexate inhibits JAK/STAT pathway and suppresses the chronic inflammatory response of the constitutively activated JAK/STAT pathway in a *Drosophila* model	38
Methotrexate	Heterochromatin	*In vivo*, adult *Drosophila* with variegated eye color phenotype Drug dose: 10 μM	Methotrexate promotes heterochromatin formation	39
Oxaliplatin (Oxal) and 5-fluoro-50-deoxyuridine (5-fluoro)	Wnt and Ras signaling pathways	*In vivo*, adult *Drosophila* Drug dose: 100 μM	The anticancer effect of oxal and 5-fluoro was demonstrated in the *Drosophila* CRC model	40
Oxazoles, thiazoles, thiazolidinedione	Wnt/Wg signaling pathway	*In vitro*, *Drosophila* cell culture Drug dose: 9 ng/μl each	CRT inhibitors efficiently block Wnt/β-catenin-induced target genes and phenotypes in various mammalian and cancer cell lines	41
Paclitaxel	PINK1	*In vivo, Drosophila* larvae Drug dose: 20 μM	Paclitaxel induces mitochondrial dysfunction in C4da neurons, and PINK1 expression suppresses the mitophagy increase induced by paclitaxel in C4da neurons	42
Sorafenib	RET-tyrosine kinase	*In vivo*, transgenic *Drosophila* at embryo, larval, and adult stages Drug dose: 1 µM	A whole-organism efficacy validation of sorafenib in the treatment of medullary thyroid carcinoma	43
Sorafenib and erlotinib; sorafenib and paclitaxel	RET-tyrosine kinase	*In vivo*, drug candidates were dissolved in DMSO and diluted in fly food (500–1000 μl)	Establishment of *Drosophila* model to identify novel therapeutic strategies focused on the multiple gene fusion oncogene KIF5B-RET	44
TAE684, crizotinib	hALK	*In vivo*, adult transgenic *Drosophila* expressing two mutations of hALK Drug dose: TAE684 (30, 50, and 100 nM) Crizotinib (250 and 500 nM)	Crizotinib and NVP-TAE684 can inhibit ALK gain-of-function mutations	45
TORC1 inhibitors	Splicing factor 2 (SF2)	*In vivo*, adult *Drosophila* Drug dose: 10 and 100 g/l	SF2 represents a highly specific therapeutic target for tumors with hyperactive TORC1 signaling	46
Trametinib and fluvastatin	Ras-PTEN	*In vivo*, transgenic *Drosophila* larvae (RNAi and GAL4-UAS lines) Drug doses: combination of 50 mM fluvastatin with 0.5 mM trametinib or with 1 mM trametinib	Establishment of the first lung cancer model (targeting Ras and/or PTEN knockdown) in *Drosophila*. Subsequent drug screening using this model revealed the potential effect of Trametinib and Fluvastatin for lung cancer	47
Trametinib and zoledronate	KRAS	*In vivo*, transgenic *Drosophila* with altered orthologs of nine colorectal cancer-related genes in the *Drosophila* hindgut was developed Drug doses: 1 µM in the fly food (each)	Establishment of *Drosophila* as a personalized cancer drug discovery platform, targeting KRAS-mutant metastatic colorectal cancer. Subsequent drug screening using this model revealed the potential effect of trametinib and zoledronate for metastatic colorectal cancer	48
Triptolide	XPB subunit of TFIIH	*In vivo, Drosophila* third instar larvae (transgenic and mutant lines) Drug dose: 100 μM	The mechanism of triptolide to induce apoptosis in p53-deficient cancer cells includes activation of the JNK death pathway	49
Vinblastine	Microtubular proteins in the mitotic spindle	*In vivo, Drosophila* larvae Drug dose: 1 μM	At low concentration, vinblastine can inhibit the dynamics of microtubule	50
Vitamin K2	ROS	*In vivo, Drosophila* larvae Drug dose: 50 mM	Vitamin K2 prevents lymphoma development by restoring mitochondrial function, which is a fundamental step in tumor treatment and VK2 strategy for cancer therapy	51
ZD6474	dRET	*In vivo*, adult transgenic *Drosophila* (eye phenotype) Drug dose: 0.2 and 1 mmol/l	ZD6474 suppresses the RET-mediated phenotype in the *Drosophila* model and ZD6474 exhibits high efficacy and very low toxicity	52
Combinations of Docetaxel-bortezomib, paclitaxel-bortezomib, paclitaxel-imatinib, paclitaxel-regorafenib	KRAS and other targets	*In silico*, *Drosophila* patient model (DPM), concentrating on *Drosophila* midgut models Drug dose: NA (in silico study)	The application of combinatorial therapy and targeted therapy demonstrated a remarkable success in the anticancer therapy (100% increase in the death of cancer cell and a complete reduction in tumorigenesis)	53

CRC, colorectal cancer; CRT, β-catenin responsive transcription; CTP, cytidine triphosphate; dRET, *Drosophila* RET orthologue; EGFR, epidermal growth factor receptor; EMS, ethyl methanesulfonate; ERK, extracellular signal–regulated kinase; GAL4-UAS system, the GAL4-upstream activating sequence system; hALK, human anaplastic lymphoma kinase; hAPC, human adenomatous polyposis coli; Hh, hedgehog; 2HMC, chalcone (E)-1-(2-hydroxyphenyl)-3-(4-methylphenyl)-prop-2-en-1-one; hTPM4, human tropomyosin 4; JAK/STAT, the Janus kinase-signal transducer and activator of transcription; KRAS, Kirsten rat sarcoma virus; l(2)gl, lethal(2)giant larvae; 4-MET, coumarin-chalcone hybrid [7-methoxy-3-(E)-3-(3,4,5-trimethoxyphenyl)acryloyl-2H-cromen-2-one]; PTEN, Phosphatase and TENsin homolog deleted on chromosome 10; RET, Rearranged during transfection; RNAi, RNA interference; ROS, reactive oxygen species; SERCA, sarco/endoplasmic reticulum Ca^2+^-ATPase.

One of the most notable features of *Drosophila* as a model organism is the availability of human-homologs cancer-related signaling pathways such as epidermal growth factor receptor (EGFR), Hedgehog (Hh), the Janus-activated kinase (JAK)-signal transducer and activation of transcription (STAT), nuclear factor κB (NF-κB), Notch, and Salvador-Warts-Hippo (SWH). These pathways have been reported to play crucial roles in the development of cancer cells in humans and are currently under intense investigation to serve as prospective targets for cancer drug discovery^9^. However, despite the continuous and intensive efforts in the identification of cancer-related genes and their associated pathways, it remains challenging to fully elucidate the global map of cancer network due to complexities in the cancer etiology and the scarcity of in-vivo platform available for conducting experimental techniques in the mammalian models^2^,^54^. Hence, the application of a simple in-vivo fruit fly model to study factors contributing to the development of cancer and how to successfully treat the cancer shall provide additional insights in the field.

*D. melanogaster* has several key qualities of model organism required to address essential yet unanswered issues of cancer drug discovery and drug repurposing, particularly at the initial phase of the study. With its minute size and fast life cycle, *Drosophila* can be maintained in a high number yet at economical cost^55^. Taking advantage on the simplicity and the versatility of the *Drosophila* cancer model, researchers have performed high-throughput validation of cancer drugs in a cost-effective, timesaving, and physiologically relevant manner. For a full list of anticancer drugs that have been successfully tested using *Drosophila* cancer model, please refer to Table 1. With such accomplishment, the application of *Drosophila* cancer model in the drug discovery and drug repurposing is open for exploration. Further efforts to screen investigative compounds with anticancer potential as well as Food and Drug Administration (FDA)-approved noncancer drugs shall be the next steps to be taken.

In general, around 68% of human cancer genes are conserved in fruit fly^9^. This highly conserved function at the level of genes and proteins between the two species suggesting that it is favorably feasible to find novel cancer drug targets in flies that are applicable to humans. This approach has been successfully applied in the discovery of novel host factors responsible in the viral propagation which later found feasible as antiviral drug target^56^. In addition, the absence of ethical restrictions to perform explorative drug testing study in a high-throughput manner in fruit flies shall offer a tremendous benefit in the anticancer drug discovery and repurposing. Hence, using a fly system, a straightforward assay to discover novel cancer-related proteins as well as a high-throughput assay to screen for new anticancer candidates from the already available FDA-approved drugs can be done in a simple and relatively economical manner.

To support cancer research and drug discovery, a continuously updated catalog of fly lines harboring mutant or transgenic genes relevant for cancer models are accessible from various fly stock centers worldwide^14^,^55^. In addition, FlyBase (flybase.org) shall provide necessary information about the use of *Drosophila* in translational research. FlyBase serves as a knowledge base that can offer an accessible and valuable entry point for researchers interested to utilize *Drosophila* models of human diseases, including cancer^55^,^57^. By taking advantage on the availability of these cancer-related fly strains and internationally recognized database, researchers will have huge opportunities to perform an in-depth analysis on the relevant genes of interest. Last, *Drosophila* is equipped with a fat body, which is analogs to the liver^58^. Drug metabolism can also occur in flies, similar to what has been reported in mammals^55^. In recent years, *Drosophila* has been shown as a prospective model organism in drug discovery and toxigenomics studies^59^–^61^, implicating that drugs and environmental toxins may be metabolized in flies in a similar fashion as in humans. With those abovementioned important signatures, fruit fly shall play a tremendous role in the anticancer drug discovery and repurposing. This simple in-vivo model will be remarkably useful in the presence budget limitations and ethical constraints of using mammalian animal models.

While much encouraging arguments has been made, a balanced view needs to be stated related to the applicability of fruit flies as a model organism in anticancer drug discovery and repurposing. Besides the various advantages of using fruit flies as a cancer model, fruit flies also have some limitations. The primary limitation of *Drosophila* is that certain cancers cannot be modeled because flies lack the corresponding organs found in humans^62^. For instance, the absence of breast, prostate, and lung tissues in flies prevents the creation of directly comparable models of these cancers. Nevertheless, *Drosophila* has been successfully used to model human epithelial and stem cell cancers. Furthermore, due to the absence of a closed circulatory system in *Drosophila*, it is difficult to model tumor-induced angiogenesis and associated changes in the tumor microenvironment^63^.

At present, the use of *D. melanogaster* in the field of oncology remains underutilized despite its vast potential. In this opinion piece, we would like to propose the prospective application of *D. melanogaster* as an in-vitro platform as well as an in-vivo model organism in the high-throughput screening of cancer drug candidates as a means of drug discovery or drug repurposing. The importance of this approach is strengthened by the fact that *Drosophila* has been widely used in the study the cancer biology and investigation of immunological signatures involved in the promotion or mitigation of cancer^7^,^8^. *D. melanogaster* shall offer justified feasibility in the investigation of novel targets for an anticancer drug. In addition, this model may serve as one of the leading model organisms used in the study of cancer biology and pharmacological approaches to treat the disease.

Comprehensive and systematic efforts at a global scale are urgently required to discover effective and economical anticancer treatments. To support this, an improved list of cancer hallmarks and oncologic-related factors responsible in the emergence of malignancy that leads to cancer-related mortality shall be the vital topics to pursue. Knowledge on those topics shall promote a great deal of improvement of pharmacotherapeutic strategies to treat cancer. In conjunction with those efforts, at the initial step of drug repurposing, *Drosophila* researchers that are equipped with vast research tools and accessible human cancer-related strains required in the high-throughput drug screening, can offer a relevant pharmacological insight. By carrying out a relatively simple drug repurposing screening assay, one may reveal new anticancer drug candidates from the list of non-anticancer FDA-approved drugs. Alternatively, one may investigate the noncanonical pharmacological effects of anticancer drugs as well as the adverse effects displayed upon the use of anticancer drug to the metazoan species in a simpler, faster, and economical ways. For example, the mechanistic basis on the neuropathic effect of paclitaxel and the anti-inflammatory effect of methotrexate (Table 1) have been demonstrated using *Drosophila* in-vivo system^42^,^64^. All results obtained in the *Drosophila* model, certainly, will be subject for translation into the mammalian models, before clinical validations in humans. In the end, while our arguments remain to be verified, we believe fruit flies will be one of our greatest allies in the discovery of novel anticancer drugs and repurposing of non-anticancer drugs in the treatment of cancer-related diseases.

## Ethical approval

Not applicable.

## Sources of funding

None.

## Authors’ contributions

F.N.: conceptualization, data curation, writing – original draft preparation, writing – reviewing and editing. E.S., M.F.A., D.C., K.D., and A.A.R.: data curation, writing – original draft preparation, writing – reviewing and editing. T.B.E.: writing – reviewing and editing, visualization, supervision.

## Conflicts of interest disclosure

The authors declare that they have no financial conflict of interest with regard to the content of this report.

## Research registration unique identifying number (UIN)

None.

## Guarantor

Talha B. Emran.

## Provenance and peer review

Not commissioned, externally peer reviewed.
